# Effects of 28-days ingestion of a slow-release energy supplement versus placebo on hematological and cardiovascular measures of health

**DOI:** 10.1186/s12970-014-0059-2

**Published:** 2014-12-10

**Authors:** Adam J Wells, Jay R Hoffman, Adam M Gonzalez, Kyle S Beyer, Adam R Jajtner, Jeremy R Townsend, Leonardo P Oliveira, David H Fukuda, Maren S Fragala, Jeffrey R Stout

**Affiliations:** Institute of Exercise Physiology and Wellness, University of Central Florida, 4000 Central Florida Blvd., Orlando, FL 32816 USA

**Keywords:** Energy supplement, Slow-release, Caffeine, Health, Safety, Comprehensive blood chemistry, Lipid profile, Complete blood counts, Resting heart rate, Blood pressure

## Abstract

**Background:**

Recently, slow release tablets have been developed to prolong energy release throughout the day. The efficacy of the delivery of slow-release caffeine alone is fairly well documented; however, an assessment of safety and tolerability of prolonged use of slow-release energy supplements is lacking. Therefore the objective of this study was to investigate the effect of daily ingestion of a slow-release energy supplement for 28 days on blood chemistry and resting cardiovascular measures in healthy men and women.

**Methods:**

Forty healthy individuals (20 males, 20 females; age: 22.73 ± 3.06 years; height: 171.68 ± 10.45 cm; mass: 74.49 ± 15.51 kg; BMI: 25.08 ± 3.66 (kg • m^2^) ^-1^) participated in this randomized, double-blind, placebo controlled study. Following a 12-hour fast, participants reported for pre-testing. Testing consisted of resting heart rate (RHR) and blood pressure (BP) measures, followed by assessment of metabolic blood chemistry, blood lipids and complete cell counts. Participants then supplemented with either Energize™ (SUPP) or placebo (PL) for 28 days. Post-testing occurred 24-hours after ingestion of the final dose and consisted of the same protocol at the same time of day as pre-testing.

**Results:**

No significant changes in outcome measures were observed. A significant difference between groups was observed for plasma glucose concentrations; however, follow-up testing revealed that pre- to post-supplementation changes were not significant for either SUPP or PL. All variables remained within normal adult reference ranges. No adverse events were reported.

**Conclusions:**

These findings indicate that 28 consecutive days ingestion of a slow release energy supplement containing caffeine in caffeine users is both safe and tolerable.

## Background

Caffeine is the most frequently consumed pharmacologically active substance in the world [1,2], where approximately 80 percent of the adult population consumes between 200–250 mg of caffeine on a daily basis [3]. Moderate doses of caffeine can lead to an increase in both physical and mental task performance [4-10], making caffeine an ideal compound for combating both fatigue and sub-optimal arousal. Caffeine’s effect is likely attributable to its function as a mild central nervous system (CNS) stimulant [11], whereby it competitively binds to adenosine receptors, leading to a suppression of its inhibitory effect on CNS activity [12]. Accordingly, caffeine is the main physiologically active ingredient in many commercially available energy supplements [13]. Nevertheless, caffeine and caffeine-containing energy supplements alike, typically induce only 90–120 minutes of increased alertness [14,15], and are often associated with an acute “crash” state following their metabolism [16,17]. Additionally, the amounts of these ingredients are often undisclosed and unregulated [18]. As a consequence, repeat administration of such energy supplements may lead to more aversive effects and dysphoric reactions over time [3]. Of particular concern are the effects of prolonged use on resting cardiovascular parameters, as well as on hepatic and renal function.

Recently, slow release tablets have been developed to prolong energy release throughout the day [14,19]. Pharmacokinetic studies have demonstrated that these tablets are able to provide a steady release of caffeine and other compounds over a longer period of time [20], likely eliminating the need for repeat administration and possibly reducing any potential adverse effects associated with repeated use [21]. Therefore, the use of these tablets to deliver an energy supplement may be beneficial for workers involved in sustained operations that demand peak cognitive and physical performance, and provide a safer alternative to repeated dosing.

The efficacy of the delivery of slow-release caffeine alone is fairly well documented [3,21-23]. However, an assessment of safety and tolerability of prolonged use of slow-release energy supplements is lacking. Therefore, the purpose of this study was to investigate the effects of 28-days ingestion of a commercially available slow-release energy supplement on blood lipid profiles, comprehensive blood chemistry, and complete blood counts in young, healthy men and women.

## Methods

### Participants

Forty healthy individuals (20 men, 20 women; age: 22.73 ± 3.06 years; height: 171.68 ± 10.45 cm; mass: 74.49 ± 15.51 kg; BMI: 25.08 ± 3.66 (kg • m^2^) ^-1^) who were regular consumers of caffeine volunteered to participate in this randomized, double-blind, placebo controlled study. Anthropometric data by group is presented in Table 1. The research protocol was approved by the New England Institutional Review Board. Following an explanation of all risks and benefits associated with the experimental protocol, each participant gave his or her informed consent to participate in this study. For inclusion in the study, participants had to be regular caffeine consumers (between 60–180 mg per day). Participants were excluded if they had any history of cardiovascular disease, metabolic, renal, hepatic, or musculoskeletal disorders or were taking any other medication (other than oral contraceptives) as determined by a confidential medical history questionnaire. Participants were also excluded as a result of any intolerance or known allergy to the supplement ingredients. While enrolled in the study, participants were permitted to maintain their normal caffeine intake.

**Table 1:** **Participant anthropometric characteristics**

**Variable**	**SUPP (n = 20)**	**PL (n = 20)**
	**(10 men, 10 women)**	**(10 men, 10 women)**
Age (years)	22.95 ± 3.05	22.5 ± 3.13
Height (cm)	172.83 ± 8.84	170.54 ± 11.97
Mass (kg)	73.77 ± 12.65	75.21 ± 18.23
BMI (kg • m^2^)^-1^	24.62 ± 3.35	25.53 ± 3.97

Data presented as mean ± SD.

### Experimental design

**Figure 1:**
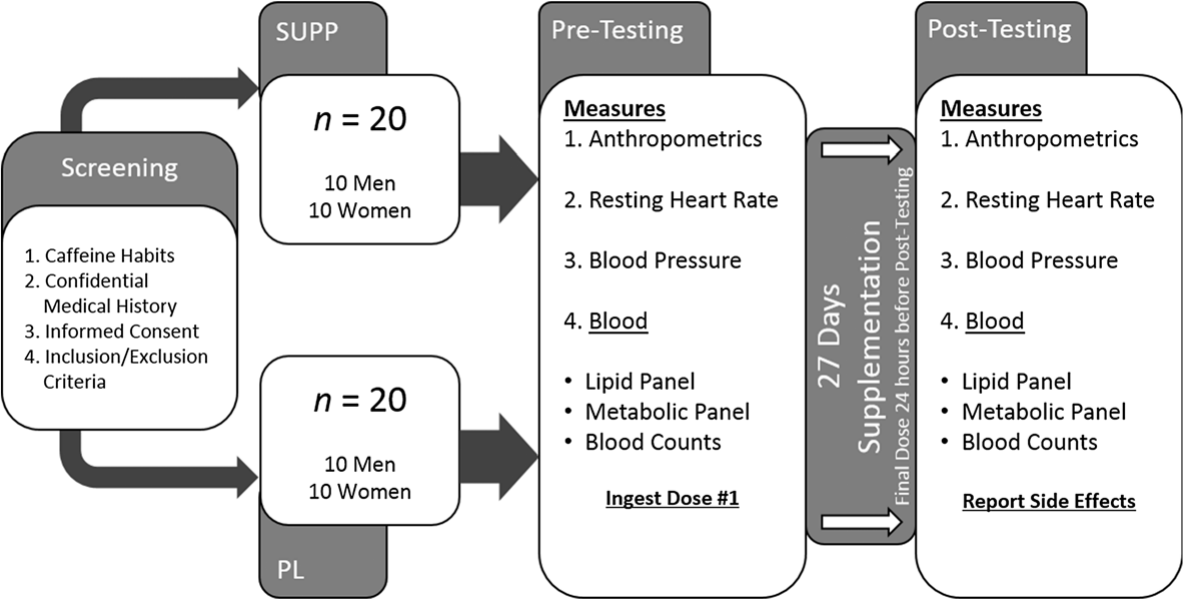
**Experimental Design Schematic.**

The experimental design is depicted in Figure 1. Following the initial screening visit, participants reported to the Human Performance Laboratory (HPL) on 22 separate occasions. Testing was conducted at pre- (visit 1) and post- (visit 22) supplementation only. Testing sessions were separated by 28 days. For each testing session, participants were required to report to the HPL following a 12-hour fast. Resting heart rate (RHR) and blood pressure (BP) were recorded, followed by a venous blood draw. Upon completion of pre-testing during visit 1, participants were required to consume the first dosage of Energize™ (iSatori, Golden, CO, USA) (SUPP) or placebo (PL) witnessed by one of the research personnel. Participants then continued supplementation for an additional 27 days. Supplement and placebo were provided by the sponsor in coded bottles. Each bottle contained 28-days’ worth of supplement or placebo. Subject numbers were assigned to each code chronologically following obtainment of consent. Subject numbers were permanently marked on their respective bottles, and each daily serving for each participant was taken from the same bottle until completion of the study. Participants were required to report to the HPL on all weekdays (20 total visits) to receive either SUPP or PL. On weekends, participants were provided 2 dosages of SUPP or PL to consume on each weekend day. Weekend supplementation was provided in zip lock bags with clear instructions on how to take the supplement. All participants were required to consume the supplement before 4 pm each day to avoid disturbance of sleep cycles and return the bags the following Monday to demonstrate adherence. Post-testing (visit 22) occurred the day following ingestion of the last dosage. Following a 12-hour fast, participants completed the same testing protocol as at pre-testing, at the same time of day. Recent reviews suggest that a daily caffeine dosage of 400 mg • d^−1^ is not associated with risk of toxicity, changes in behavior or adverse cardiovascular effects [2,24]. Since participants in the present study reported an average daily consumption of 60–180 mg of caffeine, the addition of the supplement placed average daily consumption below this threshold. As a result, changes in hematological and/or cardiovascular variables are likely related to the time-release nature of the supplement. All participants were asked to report any adverse effects they felt were directly attributable to ingestion of the supplement at post-testing.

### Testing sessions

During visits 1 and 22, anthropometrics, RHR and BP were assessed. Upon arrival to the HPL, body mass (± 0.1 kg) and height (± 0.1 cm) were measured using a Health-O-Meter professional scale (Patient Weighing Scale, Model 500 KL; Pelstar, Alsip, IL, USA). Following 15-minutes of rest in a supine position, RHR and BP were assessed using a digital blood pressure monitor (Omron Healthcare, Inc, HEM-712C, Vernon Hills, Illinois, USA). A resting blood sample was then obtained from an antecubital vein in the superficial forearm using a 21-gauge disposable needle stick by an experienced lab technician. Blood was drawn into serum separator tubes (SST), serum tubes, and EDTA tubes. SST and serum tubes were allowed to clot for 30 minutes prior to centrifugation and then separated at 3000 × g for 15 minutes at room temperature. The resulting serum from the serum tube was then transferred to a 5 mL transport tube. Samples were then packaged along with a requisition form for analysis at a commercial laboratory (Quest Diagnostics, Tampa, FL, USA) for blood lipids, metabolic blood chemistry, and complete blood counts.

### Supplement

The SUPP and PL were ingested in tablet form, and two tablets were consumed per dose. Tablets were identical in appearance and taste. The ingredients in SUPP are presented in Table 2, while the placebo consisted of only rice powder. Participants were provided one dose per day according to manufacturer recommendations. Supplementation began immediately following the blood draw at pre- and ceased the day before post-testing. Participants did not ingest supplement or placebo on the day of post-testing. Supplement administration was witnessed by research personnel during all weekdays. Additional work in our laboratory showed elevated plasma caffeine and theobromine concentrations for 8 hours following ingestion of Energize™ versus placebo, as determined via high performance liquid chromatography (HPLC) [25].

**Table 2:** **Supplement Ingredients (per serving size)**

**Ingredients**	**Amount**	
Thiamine (vitamin B1) (as thiamine hydrochloride)	5.2	mg
Vitamin B6 (as pyridoxine hydrochloride)	25	mg
Folate (as folic acid)	200	μg
Vitamin B12 (as cyanocobalamin)	3	μg
Magnesium (as magnesium oxide)	150	mg
**Proprietary Energizer Formula:**	**1600**	**mg**
L-Tyrosine		
Glucuronolactone		
Natural Caffeine (coffee arabica) (seed)-sustained release	194	mg
Theobromine (theobroma cacao) (seed)		
Rhodiola Rosea Extract (root) (standardized to contain 3% rosavins & 1% salidroside)		
Korean Ginseng Powder (root)		
Octacosonal (from sugar cane polycosanol)		

mg = milligrams; μg = micrograms.

### Statistical analyses

Statistical analysis of the data was accomplished using a 2 by 2 repeated measures analysis of variance (ANOVA) to determine between groups differences (SUPP vs. PL). In the event of significant differences between groups at baseline, data from the corresponding pre-values were used as a covariate in subsequent analysis. In the event of a significant F-ratio, dependent t-tests were used for pairwise comparisons. A criterion α-level of p ≤ 0.05 was used to determine statistical significance. Pre- and post- mean values for all blood variables were compared against clinically accepted normative data. Data are presented as mean ± SD.

## Results

Compliance among all participants was 98.3%. No significant between-group differences were observed at baseline for blood lipid variables, metabolic blood chemistry or complete blood counts. Study participants reported no adverse events during supplementation with either SUPP or PL.

## Heart rate and blood pressure measures

Pre- to post-supplementation changes for RHR and BP are presented in Table 3. No significant differences between the groups were observed for RHR, SBP or DBP (p = 0.945, p = 0.327, and p = 0.678, respectively) from pre- to post-supplementation.

**Table 3:** **Changes in resting heart rate and blood pressure**

**Variable**	**SUPP (n = 20)**		**PL (n = 20)**	
	**Pre**	**Post**	**Pre**	**Post**
Resting Heart Rate (bpm)	67.45 ± 16.70	64.40 ± 11.67	68.10 ± 9.40	64.85 ± 9.09
Systolic Blood Pressure (mmHg)	123.25 ± 10.96	120.10 ± 11.06	122.25 ± 13.01	113.15 ± 26.95
Diastolic Blood Pressure (mmHg)	70.10 ± 8.33	68.65 ± 7.47	71.25 ± 7.85	68.95 ± 5.83

Pre = pre-supplementation; Post = post-supplementation; Data presented as mean ± SD.

### Lipid panel

Pre- to post-supplementation changes in blood lipids are presented in Table 4. No significant differences between the groups were observed for total cholesterol (p = 0.523), HDL cholesterol (p = 0.235), triglycerides (p = 0.752), LDL cholesterol (p = 0.850), non-HDL cholesterol (p = 0.977) or the cholesterol to HDL ratio (p = 0.861) from pre-to post-supplementation. All values for all blood lipid measures remained within reference norms.

**Table 4:** **Changes in blood lipids**

**Variable**	**SUPP (n = 20)**		**PL (n = 20)**		**Reference Range**
	**Pre**	**Post**	**Pre**	**Post**	
Total Cholesterol (mg/dL)	163.5 ± 22.8	163.8 ± 24.1	155.5 ± 25.6	152.2 ± 25.2	125-170 mg/dL
HDL Cholesterol (mg/dL)	57.9 ± 12.3	60.4 ± 16.5	56.9 ± 16.0	55.9 ± 10.7	≥ 40^M^, ≥ 46^F^ mg/dL
Triglycerides (mg/dL)	71.0 ± 25.8	74.7 ± 30.6	70.5 ± 27.9	77.0 ± 37.6	38-152^M^, 40-136^F^ mg/dL
LDL Cholesterol (mg/dL)	91.3 ± 25.0	88.5 ± 22.4	84.7 ± 21.1	81.0 ± 23.4	<130 mg/dL (calc)
Risk Ratio (CHOL/HDL)	3.0 ± 0.8	2.9 ± 0.7	2.9 ± 0.8	2.8 ± 0.8	≤ 5.0 (calc)
Non-HDL cholesterol	105.6 ± 25.6	103.5 ± 23.5	98.7 ± 24.6	96.4 ± 28.2	n/a mg/dL (calc)

Pre = pre-supplementation; Post = post-supplementation; ^M^ = Male; ^F^ = Female; Data presented as mean ± SD.

### Metabolic blood chemistry

Pre- to post-supplementation changes in metabolic blood chemistry are presented in Table 5. A significant difference between groups was observed for plasma glucose concentrations (p = 0.028); however, follow-up testing revealed that pre- to post-supplementation changes were not significant for either SUPP (p = 0.077) or PL (p = 0.116). No other changes in any blood chemistry variables were noted. All values for the metabolic blood chemistry measures remained within reference norms.

**Table 5:** **Changes in blood chemistry**

**Variable**	**SUPP (n = 20)**		**PL (n = 20)**		**Reference Range**
	**Pre**	**Post**	**Pre**	**Post**	
Glucose	88.3 ± 5.2	85.8 ± 6.1	83.2 ± 10.2	88.1 ± 6.24 ‡	65-99 mg/dL
Urea Nitrogen (BUN)	15.1 ± 4.2	15.4 ± 4.3	16.4 ± 4.1	16.4 ± 4.39	7-20 mg/dL
Creatinine	0.9 ± 0.2	1.0 ± 0.2	0.9 ± 0.2	1.0 ± 0.21	0.60-1.35^M^, 0.50-1.10^F^ mg/dL
eGFR Non-Afr. American	107.3 ± 15.6	94.5 ± 15.8	103.6 ± 18.2	95.8 ± 14.82	≥ 60 mL/min/1.73 m^2^
eGFR Afr. American	124.4 ± 18.2	109.5 ± 18.2	119.9 ± 21.0	110.9 ± 17.19	≥ 60 mL/min/1.73 m^2^
Sodium	142.3 ± 2.9	143.2 ± 3.1	141.8 ± 3.2	143.1 ± 2.79	135-146 mmol/L
Potassium	4.6 ± 0.5	5.1 ± 0.5	4.7 ± 0.4	4.9 ± 0.57	3.8-5.1 mmol/L
Chloride	104.9 ± 2.6	105.7 ± 2.7	104.5 ± 2.2	104.9 ± 3.79	98-110 mmol/L
Carbon Dioxide	24.4 ± 2.7	25.4 ± 2.5	24.8 ± 2.0	26.1 ± 1.86	19-30 mmol/L
Calcium	9.6 ± 0.4	9.8 ± 0.3	9.7 ± 0.4	9.7 ± 0.33	8.6-10.3^M^, 8.6-10.2^F^ mg/dL
Protein (TOTAL)	7.0 ± 0.4	7.1 ± 0.4	7.2 ± 0.5	7.0 ± 0.41	6.1-8.1 g/dL
Albumin	4.5 ± 0.3	4.4 ± 0.3	4.5 ± 0.3	4.4 ± 0.28	3.6-5.1 g/dL
Globulin	2.5 ± 0.4	2.6 ± 0.3	2.7 ± 0.3	2.6 ± 0.27	1.9-3.7 g/dL (calc)
Albumin/Globulin Ratio	1.8 ± 0.4	1.7 ± 0.3	1.7 ± 0.2	1.7 ± 0.19	1.0-2.5 (calc)
Bilirubin (TOTAL)	0.8 ± 0.5	0.7 ± 0.3	0.7 ± 0.4	0.7 ± 0.41	0.2-1.1 mg/dL
Alkaline Phosphatase	67.6 ± 17.2	65.4 ± 18.8	59.4 ± 21.4	57.0 ± 21.58	40-115^M^, 33-115^F^ U/L
AST	25.9 ± 15.7	24.5 ± 12.0	20.1 ± 8.8	20.0 ± 10.2	12-32 U/L
ALT	21.4 ± 11.2	22.2 ± 13.3	17.8 ± 7.9	18.8 ± 12.63	9-46^M^, 6-29^F^ U/L

‡ = Between groups interaction, p ≤ 0.05; Pre = pre-supplementation; Post = post-supplementation; eGFR = epidermal growth factor receptor; AST = aspartate aminotransferase; ALT = alanine aminotransferase; ^M^ = Male; ^F^ = Female; Data presented as mean ± SD.

### Complete blood counts

Pre- to post-supplementation changes in complete blood counts are presented in Table 6. No significant differences between the groups were observed for any of the cell count variables. Values for all blood count variables remained within reference norms.

**Table 6:** **Changes in complete blood counts**

**Variable**	**SUPP (n = 20)**		**PL (n = 20)**		**Reference Range**
	**Pre**	**Post**	**Pre**	**Post**	
WBC Count	6.0 ± 1.4	6.4 ± 1.7	6.1 ± 1.3	6.0 ± 1.5	4.5-13.0 Thousand/uL
RBC Count	4.6 ± 0.5	4.6 ± 0.6	4.7 ± 0.6	4.7 ± 0.6	4.10-5.70^M^, 3.80-5.10^F^ Million/uL
Hemoglobin	13.7 ± 2.0	13.5 ± 2.1	13.8 ± 1.0	13.6 ± 1.5	12.0-16.9^M^, 11.5-15.3^F^ g/dL
Hematocrit	39.7 ± 7.5	41.2 ± 5.7	41.6 ± 2.6	41.1 ± 4.1	36.0-49.0^M^, 34.0-46.0^F^%
MCV	89.0 ± 5.5	89.0 ± 5.8	88.8 ± 6.8	89.1 ± 6.9	78.0-98.0 fL
MCH	29.5 ± 2.6	29.3 ± 2.4	29.5 ± 2.7	29.5 ± 2.7	25.0-35.0 pg
MCHC	33.1 ± 1.3	32.8 ± 0.9	33.2 ± 1.0	33.1 ± 0.9	31.0-36.0 g/dL
RDW	14.1 ± 1.5	14.1 ± 1.3	13.7 ± 1.0	13.9 ± 0.9	11.0-16.0%
Platelet Count	215.5 ± 47.3	222.8 ± 54.6	207.3 ± 45.9	207.6 ± 47.1	150-400 Thousand/uL
Abs Neutrophils	3403.9 ± 1131.6	3790.1 ± 1660.3	3289.9 ± 1194.9	3482.3 ± 1198.5	1800-8000 cells/uL
Abs Lymphocytes	1947.6 ± 471.3	1934.3 ± 570.4	2006.3 ± 640.6	1954.7 ± 533.1	1200-5200 cells/uL
Abs Monocytes	428.9 ± 122.0	419.2 ± 124.2	414.3 ± 109.0	388.0 ± 118.4	200-900 cells/uL
Abs Eosinophils	179.6 ± 122.2	213.0 ± 268.7	124.4 ± 98.6	131.5 ± 105.4	15-600 cells/uL
Abs Basophils	30.2 ± 19.9	28.7 ± 15.1	25.4 ± 12.5	23.8 ± 7.9	0-250 cells/uL
Neutrophils	56.0 ± 7.4	57.8 ± 11.5	57.6 ± 6.7	57.4 ± 7.4	%
Lymphocytes	33.0 ± 6.5	31.6 ± 9.8	33.1 ± 6.9	33.3 ± 7.4	%
Monocytes	7.2 ± 1.5	6.7 ± 1.5	6.9 ± 1.6	6.7 ± 1.9	%
Eosinophils	3.2 ± 2.4	3.4 ± 4.4	2.0 ± 1.4	2.2 ± 1.8	%
Basophils	0.5 ± 0.4	0.5 ± 0.4	0.4 ± 0.2	0.4 ± 0.2	%

Pre = pre-supplementation; Post = post-supplementation; WBC = white blood cell; RBC = red blood cell; MCV = mean corpuscular volume; MCH = mean corpuscular hemoglobin; MCHC = mean corpuscular hemoglobin concentration; RDW = red blood cell distribution width; Abs = absolute; ^M^ = Male; ^F^ = Female; Data presented as mean ± SD.

## Discussion

This is the first study to examine health and safety markers following prolonged daily ingestion of a slow-release energy supplement. The results of this study indicate that daily ingestion of a slow-release energy supplement containing approximately 200 mg · day^−1^ caffeine in combination with other ingredients for 28 days does not significantly affect blood lipids, metabolic blood chemistry profiles or blood counts. In addition, no changes in resting cardiovascular measures were noted, suggesting that prolonged use of the commercially available slow-release energy supplement Energize™, is apparently safe in young healthy adults.

Previous investigations have primarily focused on the efficacy of ingesting a single, moderate dose of slow release caffeine (SRC) in sleep-deprived subjects [3,10,23]. Although a number of these studies have reported SRC tolerability [21] and some have reported adverse events [21,26], studies have been limited to the acute response following a single 150–600 mg dose of SRC [3,20]. Lagarde and colleagues [20] examined a number of cardiovascular parameters including heart-rate, blood pressure and blood chemistry prior to and following the acute ingestion of 150, 300 and 600 mg of SRC in healthy male and female participants. Participants were subjected to 32-hours of sleep-deprivation in a cross-over design with a 1-week wash out period. During this time, participants underwent regular clinical evaluations, while tolerance was evaluated by complaints reported by the participants. Adverse events were reported by 8 of the 24 participants (7 female, 1 male), which included numbness, shaking, muscular pains, heart palpitations, and headaches. Two adverse events were reported in the placebo group. No significant changes in blood chemistry were observed; however, the authors did not delineate the blood tests performed. Reports of shivering and tachycardia were recorded in the 600 mg group; however, a clear break-down of adverse events by dosage was not presented. Additionally, it is not clear whether the adverse effects were related to the SRC (at any dosage), or due to the effects of sleep-deprivation.

Patat and colleagues [3] compared the effects of 600 mg SRC to placebo in a number of psychomotor and cognitive tasks in sleep-deprived subjects. Similar to our study, they utilized routine laboratory tests including complete blood counts and general blood chemistry in order to assess any potential negative effects of supplementation on resting cardiovascular parameters. In line with our findings, they observed no clinically relevant changes in routine laboratory tests and reported no safety related drop-out issues. However, in contrast to our findings, three adverse events were reported for the SRC group, including an episode of anxiety in one subject, and one episode of trembling and diarrhea in another. The observed differences between the results of the present study, and those of Lagarde et al. [20] and Petat et al. [3], is likely related to the differences in the dosage used. By comparison, we used a relatively moderate dose (approx. 200 mg SRC) compared to the larger 600 mg dose utilized by others. Lagarde et al. [20] suggest that the optimal dose, defined as the maximum effect without any side effects, is 300 mg of SRC. Considering that no adverse events were reported in the present study, our results appear to support the conclusions from Lagarde and colleagues [20]. Tolerability issues reported in previous studies appear to be transient in nature, with spontaneous remission occurring soon after the symptoms present [3,20]. This did not appear to be a concern in our study suggesting that tolerability in caffeine users is not an issue at moderate doses of SRC.

Comparable results have been observed in other studies investigating the safety aspects of similar multi-ingredient energy/thermogenic supplements [27,28]. Following 28-days ingestion of a popular multi-ingredient thermogenic drink, Roberts and colleagues [28] reported no significant group by time interactions for lipid profiles, complete blood counts or general blood chemistry in a group of sixty college aged males. Further, no significant effects were reported in resting cardiovascular measures (RHR, SBP and DBP). Similarly, Lockwood and colleagues [27] observed no significant changes in clinical safety markers following 10-weeks of daily supplementation with a low-calorie multi-ingredient energy drink. Both of these studies utilized supplements that contained similar amounts of caffeine (approx. 200 mg) along with other ingredients. The safety data in the present study appears to be consistent with that of similar energy blend supplements delivered in a non-time-released manner.

## Conclusions

In summary, this appears to be the first investigation on the health and safety aspects of prolonged ingestion of a commercially available slow-release energy supplement. A 28-day ingestion protocol resulted in no changes in lipid profile, blood chemistry, blood counts and resting cardiovascular measures, and all measures remained within normal, established reference ranges for adults. These findings indicate that 28 consecutive days ingestion of a moderate amount of caffeine, as part of a multi-ingredient time-release supplement, in caffeine users is both safe and tolerable. This may have important relevance in professions, where continuous operations demand peak cognitive and physical performance for sustained periods of time.

## Abbreviations

SRC: Slow-release caffeine
CNS: Central nervous system
SUPP: Supplement (Energize™)
PL: Placebo
HPL: Human performance laboratory
BP: Blood pressure
RHR: Resting heart rate
SBP: Systolic blood pressure
DBP: Diastolic blood pressure
EDTA: Ethylenediaminetetraacetic acid
